# Entropy-Guided Sedation Is Associated with Improved Hemodynamic Stability and Recovery During ERCP: A Prospective Real-World Cohort Study

**DOI:** 10.3390/jcm15103665

**Published:** 2026-05-10

**Authors:** Sonia Elena Popovici, Stelian Adrian Ritiu, Bogdan Miutescu, Tudor Voicu Moga, Ioan Sporea, Dorel Sandesc, Ovidiu Bedreag, Marius Păpurică, Raluca Lupusoru, Alina Popescu

**Affiliations:** 1 Faculty of Medicine, Victor Babes University of Medicine and Pharmacy, 300041 Timișoara, Romania; 2 Doctoral School, Victor Babes University of Medicine and Pharmacy, Eftimie Murgu Square 2, 300041 Timișoara, Romania; 3 Anaesthesia and Intensive Care Research Center (CCATITM), Victor Babes University of Medicine and Pharmacy, 300041 Timișoara, Romania; 4 Advanced Regional Research Center in Gastroenterology and Hepatology, Department VII: Internal Medicine II, Discipline of Gastroenterology and Hepatology, Victor Babes University of Medicine and Pharmacy, 300041 Timișoara, Romania; 5 Clinic of Anaesthesia and Intensive Care, Emergency County Hospital “Pius Brînzeu”, 300723 Timișoara, Romania; 6 Center for Modeling Biological Systems and Data Analysis, Department of Functional Science, Victor Babes University of Medicine and Pharmacy, 300041 Timișoara, Romania

**Keywords:** entropy monitoring, ERCP, procedural sedation, propofol, hemodynamic stability, recovery, electroencephalogram

## Abstract

**Background**: Sedation-related adverse events remain a concern during endoscopic retrograde cholangiopancreatography (ERCP), even when sedation is administered by anesthesiologists. Standard monitoring may not accurately reflect sedation depth. Electroencephalogram-based monitoring using Entropy provides an objective assessment of sedation depth and may optimize sedation management. **Methods**: This prospective, single-center, observational cohort study included 100 adult patients undergoing ERCP under anesthesiologist-administered sedation. Patients were allocated to two study groups: standard monitoring or advanced monitoring. The primary outcome was the incidence of sedation-related adverse events. Secondary outcomes included sedation depth, hemodynamic parameters, and recovery profiles assessed by the Aldrete score. **Results**: The overall incidence of sedation-related adverse events did not differ significantly between groups. However, the Entropy-monitored group had a significantly lower incidence of hypertensive episodes (6% vs. 26%, *p* = 0.007) and showed a trend toward fewer cardiopulmonary events. Sedation depth correlated significantly with adverse events, with deeper sedation associated with increased hemodynamic instability. Despite achieving slightly deeper sedation, patients in the Entropy group demonstrated significantly faster recovery, with higher Aldrete scores at 5 min (*p* = 0.003) and 15 min (*p* < 0.001). **Conclusions**: Entropy monitoring during anesthesiologist-administered sedation for ERCP was not associated with a significant reduction in overall adverse event incidence, a finding that should be interpreted in the context of the study’s limited statistical power (29.3%). However, it was associated with a significantly lower incidence of intraprocedural hypertension and faster postprocedural recovery, suggesting a role in optimizing sedation depth and hemodynamic stability rather than broadly reducing composite adverse events. These findings are hypothesis-generating and require confirmation in larger, adequately powered randomized controlled trials before clinical implementation can be recommended.

## 1. Introduction

Endoscopic retrograde cholangiopancreatography (ERCP) is a complex therapeutic procedure requiring adequate sedation to ensure patient comfort and procedural success. However, sedation-related cardiopulmonary complications remain a significant concern, occurring in up to 4–16% of cases, with a reported mortality of approximately 0.2% [1]. Although anesthesiologist-administered sedation improves procedural safety, adverse events remain common, particularly in high-risk patients [2]. Standard monitoring techniques rely largely on clinical assessment and may not accurately reflect sedation depth in real time [3]. Electroencephalography-based monitoring techniques, such as Entropy (GE Healthcare, Helsinki, Finland), provide an objective and continuous assessment of sedation depth [4]. Entropy-derived indices correlate with clinical sedation scales and may allow more precise titration of sedative agents. Despite these theoretical advantages, evidence regarding the clinical impact of Entropy monitoring during sedation for ERCP remains limited and inconclusive [5,6].

Depth of anesthesia monitoring using processed electroencephalogram (EEG)-based devices presents the possibility of objectively and continuously evaluating sedation depth. Entropy monitoring, which analyzes EEG data to gauge the variability in temporal brain activity, yields two principal metrics: State Entropy (SE) and Response Entropy (RE) [7]. These indices exhibit a reduction in value as sedation intensifies, demonstrating a strong correlation with established clinical sedation scales; specifically, correlation coefficients of 0.819 for RE and 0.753 for SE have been observed in relation to Observer’s Assessment of Alertness/Sedation scores [8]. Consequently, Entropy monitoring offers a dependable means of differentiating between varying degrees of propofol-induced sedation, encompassing the spectrum from wakefulness to profound sedation [9].

Theoretical benefits of Entropy monitoring lie in its capacity to facilitate the precise titration of sedative drugs, thereby maintaining an ideal sedation depth; this could potentially mitigate both oversedation, which elevates the likelihood of respiratory depression and hemodynamic instability, and undersedation, which might lead to patient distress and procedural delays [10]. A preliminary investigation assessing advanced monitoring techniques, including processed EEG, during procedural sedation revealed a statistically significant decrease in the incidence of respiratory depression events (an absolute reduction of 22.1%) and desaturation events (an absolute reduction of 24.2%) when contrasted with standard monitoring protocols [11]. However, prior evidence remains heterogeneous. A systematic review and meta-analysis of depth-of-anesthesia monitoring during procedural sedation did not show significant overall reductions in hypoxemia or hypotension, although it suggested that EEG-guided monitoring may influence sedative titration during selected interventional procedures [12]. In addition, individual studies in endoscopic settings, such as the study by Stasiowski et al. [13], have reported fewer inadequate anesthesia events with anesthesia guidance based on State Entropy, even though differences in hemodynamic variables were of limited clinical relevance.

The American Society of Anesthesiologists has observed that the existing body of research does not provide conclusive evidence regarding the impact of monitoring consciousness depth on patient outcomes during procedural sedation, notwithstanding the potential advantages [14]. Additionally, most studies on depth of anesthesia monitoring during procedural sedation have looked at diverse patient populations undergoing a range of procedures. Very few studies focus specifically on endoscopic retrograde cholangiopancreatography (ERCP), a procedure characterized by its inherent complexity, extended duration, and the requirement for prone or semi-prone positioning for the patients. The specific benefits of using Entropy monitoring during anesthesiologist-administered sedation for ERCP remain inadequately defined. Given the increased incidence of sedation-related complications associated with endoscopic retrograde cholangiopancreatography (ERCP), the expanding role of anesthesiologists in ERCP sedation protocols, and the capacity of electroencephalogram (EEG)-based monitoring to optimize sedation depth, it is crucial to evaluate the efficacy of Entropy monitoring in reducing adverse events within this specific clinical context [1,9].

The role of Entropy monitoring during anesthesiologist-administered sedation for ERCP remains insufficiently defined, particularly in relation to clinically relevant outcomes such as hemodynamic stability and recovery. Given the complexity of ERCP and the incidence of sedation-related complications, there is a need to better understand whether objective EEG-based monitoring can optimize sedation management beyond standard clinical assessment. This study aimed to evaluate the real-world clinical impact of Entropy monitoring on sedation management during ERCP. The primary outcome was the incidence of sedation-related adverse events during the procedure. Secondary outcomes included the correlation between Entropy-derived indices and clinical sedation scales; total anesthetic requirements; postprocedural recovery profile; and the relationship between sedation depth and the occurrence of adverse events [8].

## 2. Methods

### 2.1. Study Design and Setting

This was a prospective, single-center, observational cohort study carried out at the “Pius Brînzeu” Emergency County Hospital in Timișoara, Romania, between April 2023 and February 2024. Patients were allocated to study groups according to the availability of the Entropy monitoring device at the time of the procedure. This pragmatic allocation reflects routine clinical practice and was not based on patient-related characteristics or predefined clinical criteria. The study protocol was reviewed and approved by the Ethics Committee of the Doctoral School Victor Babes University of Medicine and Pharmacy Timisoara (approval number: 15, date: 15 March 2022). Written informed consent was obtained from all participants before their enrollment in the study. All procedures were carried out in accordance with the ethical principles of the Declaration of Helsinki (2013 revision). ERCP procedures were performed by one of three senior endoscopists, each with over 15 years of experience in advanced therapeutic endoscopy, ensuring a high level of consistency in both procedural technique and clinical management throughout the study period.

### 2.2. Study Population

One hundred consecutive adult patients (aged 22–88 years) scheduled for elective endoscopic retrograde cholangiopancreatography (ERCP) under procedural sedation were screened for eligibility. Patients were eligible for inclusion if they met all the following criteria: (1) adult patients aged ≥18 years; (2) scheduled for elective ERCP; (3) planned anesthesiologist-delivered sedation; (4) American Society of Anesthesiologists (ASA) physical status classification I–III; and (5) ability to provide written informed consent. Exclusion criteria included: (1) known hypersensitivity or contraindication to any study medication components; (2) known neurological conditions that could interfere with EEG monitoring (e.g., active seizure disorder, recent stroke, severe dementia); (3) emergency ERCP requiring immediate intervention; (4) general anesthesia with endotracheal intubation planned before procedure; (5) ASA physical status IV or higher; (6) pregnancy or lactation; (7) anticipated difficult airway (Mallampati class IV or history of difficult intubation); (8) inability to provide informed consent or refusal to take part in the study.

Patients included in the study were divided into two groups according to the monitoring strategy used during sedation. The Entropy group (Group 1) received the same standard monitoring in addition to electroencephalographic depth-of-sedation monitoring using Entropy—State Entropy (SE) and Response Entropy (RE) derived from a GE CARESCAPE B650 monitor (GE Healthcare, Helsinki, Finland). The standard monitoring group (Group 2) received routine intra-procedural monitoring, including continuous electrocardiography, non-invasive blood pressure measurement, pulse oximetry, and, when available, capnography. When Entropy monitoring was not available, patients were managed using standard monitoring alone. Although allocation was not randomized and depended on device availability, baseline characteristics were comparable between groups, suggesting a balanced distribution of patient-related factors.

### 2.3. Sedation and Monitoring Protocol

Sedation was based on a propofol regimen, with dosing titrated to achieve adequate procedural conditions while maintaining cardiorespiratory stability. Sedation was delivered in the dedicated ERCP suite equipped with standard and advanced monitoring, resuscitation equipment, and an anesthesia machine. After the patient arrived in the ERCP suite, peripheral intravenous access (18/20 G cannula) was established, and an infusion of balanced crystalloid (Plasmalyte, Baxter Healthcare, Lessines, Belgium) was started intravenously, with a total target dose of 10 mL/kg. Supplemental oxygen (2–4 L/min) was administered to all patients throughout the procedure via nasal cannula.

All patients received standard monitoring before the induction of sedation, including continuous electrocardiography (ECG), non-invasive blood pressure measurement of systolic blood pressure (SBP), diastolic blood pressure (DBP), and mean arterial pressure (MAP), and pulse oximetry. Capnography was used when technically available; however, given its inconsistent application across procedures, it was not considered a standardized component of the monitoring protocol, and its use was not systematically recorded. This is acknowledged as a limitation of the current study. The level of sedation was assessed clinically using the Modified Observer’s Assessment of Alertness/Sedation (MOAA/S) score in both groups. In patients assigned to the Entropy group, sedation depth was primarily guided by State Entropy obtained from a GE Healthcare CARESCAPE B650 monitor (GE Healthcare, Helsinki, Finland), with the MOAA/S score used as a secondary confirmatory safety measure. Three Entropy sensors were applied to the patient’s forehead prior to sedation, and baseline values were recorded while patients were awake to ensure adequate signal acquisition and electrode impedance.

In the Entropy group (Group 1), sedation titration was driven by State Entropy (SE) values according to a predefined protocol. This approach was standardized prior to the study through structured training of all participating anesthesiologists and supported by a written reference card available in the ERCP suite.

The titration algorithm was as follows: when SE exceeded 80 in a patient showing clinical signs of inadequate sedation, a propofol bolus of 0.5 mg/kg was administered; when SE was within the target range of 60–80 with clinically adequate sedation, no additional bolus was given; when SE fell below 60, further propofol administration was withheld, even in patients appearing clinically comfortable (MOAA/S 2–3); and when hemodynamic instability suggestive of oversedation occurred alongside SE < 60, propofol was withheld and the event was managed accordingly.

A predefined safety override ensured that in the event of hemodynamic compromise, patient safety took absolute priority over Entropy targets regardless of SE values. These elements confirm that Entropy monitoring functioned as the primary driver of sedation titration, rather than as a passive monitoring modality.

The target level of sedation was moderate to deep sedation, sufficient to ensure patient immobility and procedural conditions while maintaining cardiorespiratory stability. In the standard monitoring group (Group 2), sedation titration was based exclusively on clinical assessment using the MOAA/S score, hemodynamic parameters, and clinical judgment.

Patients were positioned in the left lateral decubitus, semi-prone position for the entire duration of the procedure. After positioning, patients received premedication with intravenous midazolam 10–20 µg/kg (Midazolam Hypericum, 5 mg/mL, Laboratoire Aguettant, Lyon, France), given there were no contraindications, and fentanyl as a bolus of 1 mcg/kg (Fentanyl Kalceks, 50 mcg/mL, Akciju sabiedrība Kalceks, Riga, Latvia). Induction of sedation was achieved by a propofol bolus of 0.5 mg/kg (Propofol MCT/LCT, 10 mg/mL, Fresenius Kabi, Bad Homburg, Germany), followed by intermittent boluses administered at 2 min to achieve the target sedation level as defined per MOAA/S scale (MOAA/S 3) or Entropy values (SE < 70). During maintenance, additional propofol boluses of 0.5 mg/kg were administered in both groups to maintain sedation targets. Ketamine (Calypsol 50 mg/mL, Gedeon Richter Ltd., Budapest, Hungary) was used only occasionally as a rescue analgesic adjunct—in a minority of patients and at low doses—when clinically indicated by signs of inadequate analgesia and was not a routine component of the sedation regimen. In case of adverse events, the management protocol included a bolus dose of 10 mg ephedrine (ephedrini hydrochloridum, 50 mg/mL, Zentiva Romania, Bucharest, Romania) for hypotension (MAP < 60 mmHg) and a bolus dose of 0.5 mg atropine (sulfat de atropina, 1 mg/mL, Takeda, Berlin, Germany) for bradycardia (HR < 45 bpm). In patients with inadequate analgesia defined as an increase in systolic blood pressure (SBP) and/or heart rate (HR) >20% from baseline, an additional bolus of fentanyl 0.5 mcg/kg was given intravenously, targeting normalization of hemodynamic status. After the end of the procedure, patients were monitored until they reached an Aldrete score of 9 or higher, followed by their discharge on the gastroenterology ward.

### 2.4. Outcome Measures

The primary outcome was the occurrence of sedation-related adverse events during ERCP, and to evaluate whether the use of Entropy monitoring was associated with a reduced incidence of these events compared to standard monitoring. Sedation-related adverse events were defined as the occurrence of one or more of the following cardiovascular or respiratory complications: hypotension—defined as a decrease in mean arterial pressure (MAP) to <65 mmHg or a reduction of ≥20% from baseline and/or the need for vasopressor administration (ephedrine); hypertension—defined as an increase in systolic blood pressure to ≥140 mmHg or an increase of ≥20% from baseline; bradycardia—defined as heart rate (HR) <50 beats per minute (bpm); tachycardia—defined as HR > 100 bpm; and desaturation—defined as peripheral oxygen saturation <90%. Sedation-related adverse events were recorded as counts of individual episodes per patient but analyzed as binary outcomes—defined as the occurrence of at least one episode of each event type during the procedure—for all between-group comparisons and multivariable analyses. This approach was prespecified and is consistent with published literature on procedural sedation outcomes.

Secondary outcomes were defined to further characterize the clinical impact of Entropy monitoring and were prioritized as follows: the correlation between Entropy-derived sedation depth indices (minimum and mean State Entropy values) and clinically assessed sedation depth using the Modified Observer’s Assessment of Alertness/Sedation (MOAA/S) score; total anesthetic requirements, including total propofol dose administered; postprocedural recovery profile, evaluated by time to awakening and Aldrete scores at 5 and 15 min; and the relationship between sedation depth and the occurrence of adverse events.

### 2.5. Statistical Analysis

Statistical analysis was performed using MedCalc Statistical Software version 20 (MedCalc Software Ltd., Ostend, Belgium). The distribution of continuous variables was assessed for normality using graphical and analytical methods. Data are presented as median with interquartile range (IQR), number and percentage, or mean ± standard deviation (SD), as appropriate.

Between-group comparisons were performed using the Mann–Whitney U test for non-normally distributed variables and Student’s *t*-test for normally distributed variables. Categorical variables are expressed as counts and percentages and were compared using the chi-square test or Fisher’s exact test, as appropriate. Associations between continuous and categorical variables were evaluated using Spearman’s rank correlation coefficient. Multivariable logistic regression analysis was conducted to explore potential independent predictors of adverse events, with results reported as odds ratios (OR) with 95% confidence intervals (CI). A two-tailed *p*-value < 0.05 was considered statistically significant. No formal a priori sample size calculation was performed; the sample size was based on patient availability during the study period. Post hoc power calculations demonstrated that the study achieved 29.3% power for the primary outcome (overall adverse event incidence: 42% vs. 56%, *n* = 50 per group), indicating substantial underpowering for this comparison, with approximately 398 patients required for 80% power. In contrast, adequate power was confirmed for intraprocedural hypertension (6% vs. 26%, power 80.9%) and delayed recovery (46% vs. 74%, power 84.7%). Two additional logistic regression analyses were performed: the first with intraprocedural hypertension as the dependent variable, including monitoring group, procedure duration, and chronic hypertension history as primary covariates, with a prespecified sensitivity analysis additionally adjusting for cannulation attempts, IECA use, and calcium channel blocker use; the second with intraprocedural hypotension as the dependent variable, including monitoring group, procedure duration, and total propofol dose.

## 3. Results

### 3.1. Study Population and Baseline Characteristics

A total of 100 patients undergoing ERCP under anesthesiologist-administered sedation were included in the analysis, with 50 patients in each study group. Baseline demographic, clinical, and procedural characteristics were well balanced between the Entropy and standard monitoring groups, with no statistically significant differences observed. This supports the comparability of the groups and reduces the likelihood that observed differences in outcomes were driven by baseline confounding. In particular, no differences were observed in age, ASA status, or comorbidities, which are known to influence sedation-related outcomes. Cardiovascular medication use was comparable between groups. IECA use was recorded in 24 patients (48%) in the Entropy group and 22 patients (44%) in the standard group (*p* = 0.84), and calcium channel blocker use was similarly balanced (10 patients [20%] vs. 12 patients [24%], *p* = 0.63). These findings confirm that the differential hemodynamic responses observed between groups cannot be attributed to differences in baseline antihypertensive medication exposure. Procedural complexity surrogates were broadly balanced between groups. The proportion of patients requiring one additional cannulation attempt was numerically higher in the standard group (38 patients [76%] vs. 29 patients [58%]), though this difference did not reach statistical significance (*p* = 0.056). No patient in either group required more than two total cannulation attempts. Procedural complications were similarly distributed between groups: bleeding occurred in 3 (6%) vs. 2 (4%) patients (*p* = 1.00), perforation in 1 (2%) vs. 2 (4%) patients (*p* = 1.00), and purulent bile in 19 (38%) vs. 13 (26%) patients (*p* = 0.18) in the Entropy and standard groups, respectively. The overall incidence of sedation-related adverse events did not differ significantly between groups. However, the Entropy-monitored group demonstrated a significantly lower incidence of hypertensive episodes (6% vs. 26%, *p* = 0.007), along with a trend toward fewer cardiopulmonary events overall.

Table 1 summarizes the baseline demographic, clinical, and procedural characteristics of the study population. Sedation requirements were comparable between groups. The total propofol dose was slightly lower in the Entropy group, although this difference was not clinically significant. Similarly, fentanyl, midazolam, and ketamine doses were broadly similar across groups. A significant difference was observed in procedure duration, which was shorter in the Entropy group compared to the standard group (median 20.0 vs. 25.0 min, *p* < 0.001). Patients in Group 2 (standard) exhibited a substantially higher incidence of intraprocedural hypertension compared to Group 1 (Entropy) (26% vs. 6%, *p* = 0.007).

### 3.2. Primary Outcome: Sedation-Related Adverse Events

**Figure 1:**
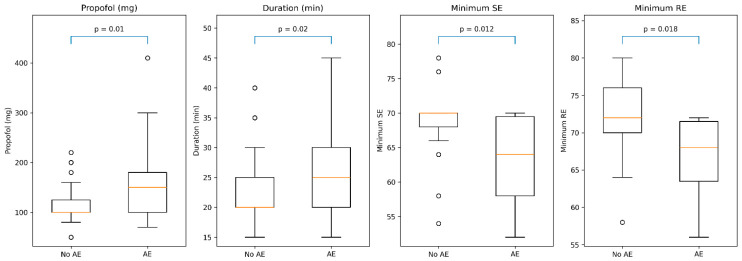
Comparison of key clinical and Entropy parameters between patients with and without adverse events. Boxplots show total propofol dose, procedure duration, minimum State Entropy (SE), and minimum Response Entropy (RE) in patients with adverse events (AE) versus those without adverse events (No AE). Patients with adverse events had significantly higher propofol requirements and longer procedures, together with lower minimum SE and RE values, consistent with deeper sedation. Boxes represent the interquartile range, horizontal lines indicate medians, whiskers indicate range, and *p*-values are shown above each comparison.

As shown in Figure 1 and illustrated in Table 2, patients who developed adverse events had higher propofol requirements and longer procedure duration compared to those without adverse events (*p* = 0.01 and *p* = 0.02, respectively). In addition, Entropy-derived indices were lower in the adverse event group (*p* = 0.012 and 0.018).

Table 3 details the correlation analysis between the objective EEG-based indices and the subjective clinical assessment. A statistically significant positive correlation was observed between minimum State Entropy (SE) values and minimum MOAA/S scores (r = 0.46, *p* < 0.001). A negative correlation was observed between minimum SE values and hypotension (r = −0.28, *p* = 0.04). Similarly, non-significant correlations were observed between minimum SE values and desaturation, bradycardia, and tachycardia (*p* > 0.05).

There was a strong negative correlation between the dose of propofol and the minimum Entropy values, indicating that higher propofol doses were associated with deeper levels of sedation. No significant correlations were found for fentanyl or ketamine. The difference between Response Entropy and State Entropy (RE–SE) remained below 10 in all patients.

**Figure 2:**
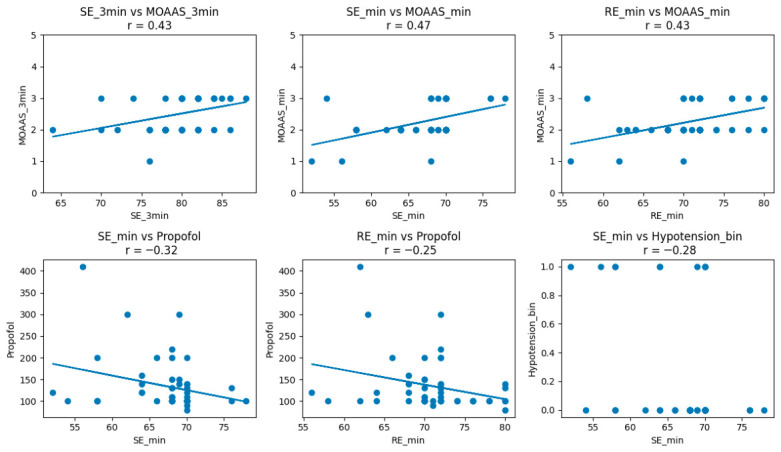
Correlation between Entropy-derived indices and clinical variables in the Entropy group.

Figure 2 shows scatter plots illustrating the relationship between State Entropy (SE), Response Entropy (RE), and sedation depth assessed by the Modified Observer’s Assessment of Alertness/Sedation (MOAA/S), as well as propofol dose and hypotension. MOAA/S scores are presented on a scale from 0 (no response) to 5 (fully alert). Hypotension is coded as a binary variable (0 = no, 1 = yes). Spearman’s correlation coefficients (r) are shown for each panel. SE and RE values were positively correlated with MOAA/S scores and negatively correlated with propofol dose and hypotension.

**Figure 3:**
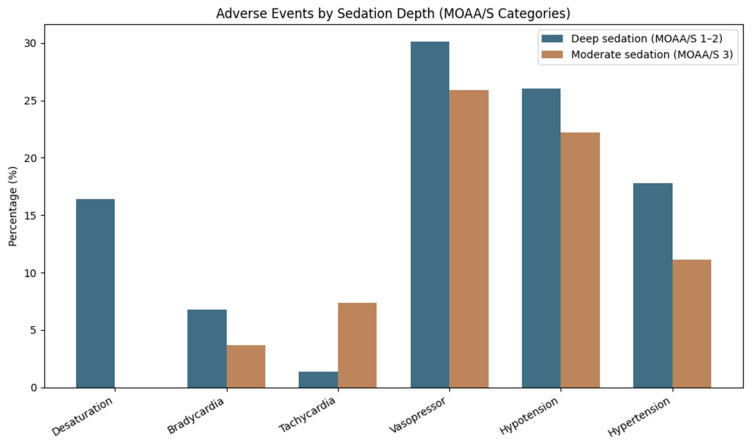
Adverse events according to sedation depth categories. Grouped bar chart showing the percentage of adverse events in patients undergoing deep sedation (MOAA/S 1–2) and moderate sedation (MOAA/S 3).

Within the Entropy group, patients were divided into two categories based on their minimum State Entropy (SE) values: SE < 60 (deeper sedation) and SE > 60 (moderate sedation). During the procedure, 7 patients (14.0%) recorded SE values less than 60, whereas 43 patients (86.0%) had SE values of 60 or higher. Patients with SE < 60 had a higher incidence of cardiovascular adverse events compared to those with SE ≥ 60. Hypotension occurred in 57.1% of cases in the deep sedation group, while it occurred in only 14.0% of cases in the moderate sedation group. The incidence of cardiovascular adverse events was higher in patients with deeper levels of sedation; minimum SE and minimum RE did not differ according to the occurrence of these events [desaturation (*p* = 0.30 and *p* = 0.74), bradycardia (*p* = 0.20 and *p* = 0.66), tachycardia (*p* = 0.42 and *p* = 0.67), hypotension (*p* = 0.20 and *p* = 0.22), or hypertension (*p* = 0.64 and *p* = 0.60)]. In the same subgroup, age was not significantly correlated with minimum SE (r = −0.14, *p* = 0.30) or minimum RE (r = 0.009, *p* = 0.95), and it was not associated with adverse events (Figure 3).

Propofol dose was analyzed in relation to sedation-related adverse events. Patients who developed hypotension received higher total propofol doses. No significant association was found between propofol dose and the occurrence of desaturation, bradycardia, tachycardia, or hypertension. Propofol dose was identified as an independent predictor of intraprocedural hypotension (OR 1.015 per mg, 95% CI 1.003–1.028, *p* = 0.012), while monitoring group was not independently associated with hypotension after adjustment (OR 0.435, 95% CI 0.140–1.354, *p* = 0.151).

Adverse events were more frequent in the standard monitoring group compared to the Entropy-monitored group (56% vs. 42%); this difference did not reach statistical significance (*p* = 0.16). Pulmonary disease was not significantly associated with desaturation in the overall cohort (Fisher’s exact *p* = 0.22) or in the Entropy subgroup (*p* = 0.15). No comparison was possible in the standard group because no patient had pulmonary disease.

In multivariable logistic regression analysis including age, procedure duration, propofol dose, minimum SE, and deep sedation, no independent predictors of adverse events were identified. Minimum SE (OR 0.99, 95% CI 0.88–1.11, *p* = 0.86) and procedure duration (OR 1.06, 95% CI 0.95–1.20, *p* = 0.29) were not associated with adverse events.

Given that intraprocedural hypertension was the primary significant outcome differentiating the two groups, an additional logistic regression analysis was performed with hypertension as the dependent variable. In the primary model, including monitoring group, procedure duration, and chronic hypertension history as covariates, Entropy monitoring was an independent significant predictor of reduced intraprocedural hypertension (OR 0.210, 95% CI 0.052–0.848, *p* = 0.028). Procedure duration (OR 1.018, 95% CI 0.944–1.098, *p* = 0.638) and chronic hypertension history (OR 0.832, 95% CI 0.270–2.565, *p* = 0.749) were not independently associated with the outcome. In a prespecified sensitivity analysis additionally adjusting for cannulation attempts, IECA use, and calcium channel blocker use, the direction and magnitude of the monitoring group effect were preserved (OR 0.191), though statistical significance was not maintained (*p* = 0.055), consistent with the limited statistical power of this model given 16 events across six covariates (events per variable = 2.7).

### 3.3. Sedation Depth and Recovery

Both cohorts started from a fully awake baseline state (median MOAA/S of 5.0). Following induction, the Entropy group (Group 1) showed a consistent decrease in cortical activity, with a median State Entropy (SE) of 70.0 at 3 min and a median minimum SE of 54.0 recorded during the procedure.

When comparing the clinical sedation scales, the minimum MOAA/S score was significantly lower in the Entropy group compared to the standard group (median 2.0 vs. 2.5, *p* = 0.04).

**Figure 4:**
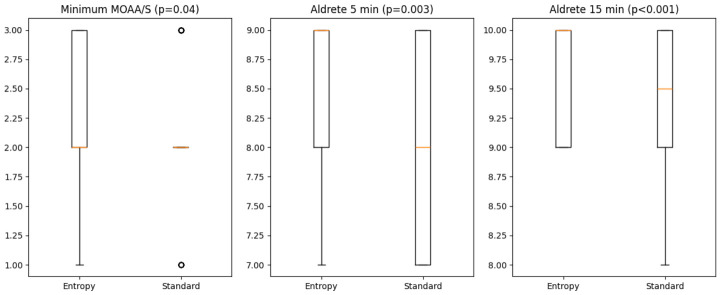
Comparison of sedation depth and recovery profile between Entropy monitoring and standard clinical assessment. Boxplots illustrate differences in minimum MOAA/S score and postprocedural recovery (Aldrete score at 5 and 15 min) between the Entropy-guided group and the standard monitoring group. The Entropy-guided group showed lower minimum MOAA/S scores and higher Aldrete scores at 5 and 15 min. Boxes represent interquartile ranges, with medians shown as horizontal lines.

Patients in the Entropy group exhibited faster emergence, as reflected by higher Aldrete scores at 5 and 15 min. As shown in Figure 4 and Table 4, Aldrete scores were higher in the Entropy group at 5 min (median 9.0 vs. 8.0, *p* = 0.003) and 15 min post-procedure (median 10.0 vs. 9.5, *p* < 0.001). At 15 min, the majority of the patients in the Entropy group had reached maximum recovery. Baseline recovery scores (Aldrete at 0 min) were similar between groups. Delayed recovery was less frequent in the Entropy-monitored group (46% vs. 74%, *p* = 0.008).

In the Entropy subgroup, end-of-procedure SE and RE values were not correlated with Aldrete scores at 5 or 15 min (*p* > 0.05). Minimum MOAA/S scores were also not correlated with recovery outcomes (*p* > 0.05).

## 4. Discussion

This prospective observational study examined the real-world clinical impact of Entropy monitoring during anesthesiologist-administered sedation for ERCP. The primary outcome—the overall incidence of sedation-related adverse events—did not differ significantly between the Entropy-monitored and standard monitoring groups (42% vs. 56%, *p* = 0.16). This non-significant result should be interpreted as an underpowered finding rather than as evidence of no effect. Notwithstanding this limitation, Entropy monitoring was associated with a significantly lower incidence of intraprocedural hypertensive episodes and a faster recovery profile. A significant correlation between Entropy values and clinical sedation depth further supports the validity of the monitoring approach. These secondary findings are hypothesis-generating and require confirmation in larger, adequately powered randomized studies. These findings suggest that the primary benefit of Entropy monitoring lies in optimizing sedation management. By providing continuous, objective feedback on sedation depth, Entropy monitoring may allow more precise titration of sedative agents, helping to avoid both oversedation and undersedation. This, in turn, may contribute to improved hemodynamic stability and more efficient recovery, even in the absence of a measurable reduction in overall adverse events. It should be noted, however, that the precise mechanism by which Entropy monitoring contributed to these differences cannot be fully established from this observational data, and the possibility that unmeasured differences in clinical practice between groups contributed to the observed outcomes cannot be entirely excluded.

The statistically significant positive correlation between minimum State Entropy (SE) and minimum MOAA/S scores (r = 0.46, *p* < 0.001) supports the use of Entropy monitoring as an objective measure of sedation depth during ERCP. This finding aligns with prior validation studies, which have reported Spearman correlation coefficients between Entropy values and OAA/S scores ranging from 0.753 to 0.819. Additionally, prediction probabilities for distinguishing different sedation levels have been found to be between 0.731 and 0.889 [15,16]. A recent prospective study involving older ERCP patients identified a more powerful correlation between the Patient State Index (PSI, another EEG-based monitor) and MOAA/S scores (ρ = 0.742, *p* < 0.001), with a median PSI of 50 (95% CI: 48–52) indicating sufficient sedation (MOAA/S 1–2) [17].

Anesthesia protocols that use Entropy usually aim for SE values between 40 and 60 for general anesthesia and between 60 and 80 for moderate sedation [18,19]. Moreover, the median minimal SE of 54.0 found in our study aligns with deep sedation. This level is often needed during complex endoscopic retrograde cholangiopancreatography (ERCP) procedures, which require the patient to be still.

This correlation is clinically relevant and reinforces its role as an objective measure of sedation depth. The small difference between RE and SE values may suggest adequate analgesia during the procedure.

Our findings should also be interpreted in the context of previous endoscopic studies evaluating EEG-guided anesthesia or sedation. Most of the literature has examined bispectral index (BIS) monitoring, which, like Entropy, derives indices from processed EEG signals. A randomized controlled trial found that BIS-guided propofol sedation for ERCP was associated with reduced propofol consumption [20]. The EndoBIS randomized trial, also conducted in ERCP patients, found no significant difference in cardiopulmonary complications, propofol dose, or recovery between BIS-guided and standard arms [21]. A study evaluating anesthesia guidance based on State Entropy during colonoscopy reported fewer inadequate anesthesia events compared with standard practice, while differences in hemodynamic variables, although statistically significant, were considered of limited clinical relevance [13]. These findings support the concept that EEG-based monitoring may primarily improve sedation titration and procedural conditions rather than consistently reducing major cardiopulmonary adverse events. This interpretation is in line with our results, where Entropy monitoring was associated with improved hemodynamic stability and faster recovery but not with a significant reduction in overall adverse event rates.

A significant finding of our research is the identified correlation between sedation depth and the incidence of adverse outcomes. The correlation between extended procedure time and higher propofol dosages with a rise in adverse events, as well as lower minimum SE and RE values, underscores the relationship between sedation depth and patient safety, with higher propofol doses being associated with increased hemodynamic instability.

The statistically significant negative correlation observed between minimum State Entropy (SE) and hypotension (r = −0.28, *p* = 0.04) suggests that deeper levels of sedation are associated with a higher incidence of hemodynamic instability. These findings align with the known cardiovascular depressant effects of propofol, thereby underscoring the necessity of preventing excessive sedation depth [5]. Consequently, they support the notion that sedation depth, rather than the monitoring method alone, is a crucial factor in cardiopulmonary instability during ERCP.

A further observation from this study refers to the reduction in intraprocedural hypertension incidence in the Entropy-guided study group (6% vs. 26%; *p* = 0.007). From a clinical perspective, intraprocedural hypertension may reflect inadequate sedation depth or increased procedural stress, which can potentially lead to patient discomfort and technical challenges during ERCP. Although procedural interruptions or technical success rates were not formally assessed in our study, it is plausible that improved hemodynamic stability in the Entropy-monitored group may be associated with better procedural conditions. Future studies incorporating procedure-related outcomes and patient-reported measures are needed to further explore this relationship.

This finding suggests that advanced monitoring may improve titration of sedation depth and mitigate procedural stress responses, with potential implications for patient safety, especially among elderly individuals and those with cardiovascular comorbidities. The reduced incidence of hypertensive episodes is clinically relevant in the context of ERCP, as patient movement or straining during critical maneuvers (such as cannulation or sphincterotomy) could increase the risk of procedure-related complications. The potential contribution of the borderline difference in cannulation attempts (58% vs. 76%, *p* = 0.056) to procedural complexity and hemodynamic stress was specifically examined in a multivariable analysis with hypertension as the dependent variable. The monitoring group remained an independent predictor of hypertension after adjustment for procedure duration, hypertension history, and cannulation attempts, supporting the interpretation that the observed reduction in hypertensive episodes reflects the active role of Entropy-guided titration rather than differences in procedural complexity alone. Furthermore, the numerically higher rate of purulent bile in the Entropy group (38% vs. 26%, *p* = 0.18)—a marker of biliary obstruction with infection and greater disease severity—suggests that if anything, the Entropy group presented with slightly more complex disease at baseline, yet still demonstrated superior hemodynamic stability, further supporting the clinical relevance of the monitoring strategy.

Our study also showed a lower incidence of bradycardia in the Entropy group (4.0% vs. 8.0%), although this difference was not statistically significant, suggesting that Entropy monitoring may help avoid excessive sedation depth. Similar findings have been reported in surgical populations, where Entropy-guided anesthesia has been associated with improved hemodynamic stability and reduced incidence of hypotension and bradycardia compared to standard monitoring [22].

Deep sedation, as assessed by a Modified Observer’s Assessment of Alertness/Sedation (MOAA/S) score of 1–2, was notably more prevalent among patients who experienced adverse events. Furthermore, desaturation episodes were significantly more frequent in the deep sedation cohort compared to those receiving moderate sedation (16.4% versus 0%, *p* = 0.02). This observation is consistent with existing registry data, indicating a correlation between deeper sedation levels during endoscopic procedures and an increased risk of respiratory complications [23].

A key clinical finding of this study was the improved recovery profile in the Entropy-monitored group. Despite achieving deeper sedation levels, patients within this group exhibited a more rapid recovery, evidenced by higher Aldrete scores at both 5 and 15 min, alongside a lower incidence of delayed recovery.

An important aspect to consider when interpreting these findings is the difference in procedure duration between the study groups. The significantly longer procedures observed in the standard monitoring group may have contributed to delayed recovery, potentially through increased cumulative exposure to sedative agents or procedural stress. Therefore, the observed differences in Aldrete scores should be interpreted with caution, as procedure duration may represent a confounding factor influencing recovery dynamics.

In addition, postoperative nausea and vomiting (PONV) represents one of the most common anesthesia-related adverse events and has a significant impact on patient satisfaction and quality of recovery, particularly in procedures requiring moderate to deep sedation. Although ERCP procedures are typically longer and may involve higher cumulative doses of sedative agents, PONV was not systematically assessed in our study. Future studies incorporating validated risk scores, such as the Apfel score, could provide additional insights into the impact of sedation strategies on postoperative recovery quality.

This finding may be partially explained by the pharmacokinetic properties of propofol. Precise titration of sedation depth may prevent excessive drug accumulation, thereby allowing faster recovery despite achieving adequate sedation levels, although the influence of procedural duration cannot be excluded [24].

In this context, Entropy monitoring enhances precision during sedation by providing continuous, objective feedback regarding cortical activity. This allows clinicians to adjust propofol dosing to the minimum effective concentration necessary for sufficient sedation at each moment throughout the procedure. In contrast, standard monitoring is based on intermittent clinical assessment, which can be delayed relative to the actual sedation depth, potentially resulting in either overshooting the target (leading to drug accumulation and extended recovery) or undershooting (necessitating rescue boluses that induce depth fluctuations and hemodynamic instability). Entropy monitoring may aid clinicians in using propofol more efficiently by avoiding both under- and over-sedation and improving recovery dynamics. This finding is consistent with the hypothesis that Entropy monitoring does not appear to directly reduce adverse events but rather influences sedation behavior, which may in turn impact hemodynamic stability.

From a clinical perspective, these findings suggest that Entropy monitoring may help refine sedation strategies during ERCP by enabling more precise titration of sedative agents. This may contribute to more stable hemodynamic conditions and improved recovery dynamics, even in the absence of a significant reduction in overall adverse events. Such benefits are particularly relevant in elderly and high-risk patients undergoing complex endoscopic procedures.

This study has several limitations that should be considered when interpreting the findings. First, the non-randomized design may introduce selection bias and limits the ability to establish causal relationships. However, group allocation was primarily determined by the availability of the Entropy monitoring device rather than patient-related or clinical factors, reflecting a pragmatic, real-world approach. In addition, baseline demographic, clinical, and procedural characteristics were well balanced between groups, supporting the internal validity of the comparisons.

Nevertheless, residual confounding cannot be excluded, and the findings should be interpreted as hypothesis-generating.

Second, the relatively small sample size and the absence of a formal sample size calculation may have limited the statistical power of the study, particularly in detecting differences in the overall incidence of adverse events. Therefore, the study may have been underpowered to detect smaller but clinically relevant differences between the two monitoring strategies.

Third, sedation was administered by approximately 20 anesthesiologists rotating monthly through the ERCP suite over the one-year study period. Although all received identical protocol training and had access to a written titration reference card, individual differences in clinical experience and algorithm interpretation cannot be excluded as a source of variability. Capnography was applied inconsistently due to variable equipment availability and was not systematically recorded. Additionally, while a structured SE-guided titration algorithm was applied in the Entropy group, contemporaneous SE and MOAA/S values were not recorded at each bolus decision point, limiting post hoc verification of protocol adherence.

A further limitation relates to the absence of formal prospective ERCP complexity grading. Although procedural complexity was partially characterized through diagnosis category, number of cannulation attempts, procedural complication rates, and procedure duration—all of which were broadly balanced between groups—a validated complexity grading system such as the ASGE classification was not prospectively applied. The borderline difference in the proportion of patients requiring a second cannulation attempt (58% vs. 76%, *p* = 0.056) cannot be entirely excluded as a contributing factor to between-group differences in procedural duration and hemodynamic outcomes and was therefore included as a covariate in the supplementary logistic regression for hypertension. Future studies should incorporate prospective ASGE complexity grading to allow more precise adjustment for procedural difficulty.

Another limitation of this study is the lack of systematic assessment of postoperative nausea and vomiting (PONV), which represents a clinically relevant outcome influencing postoperative recovery and patient satisfaction. The absence of PONV evaluation, including risk stratification using validated tools such as the Apfel score, limits the ability to fully characterize the impact of sedation strategy on overall recovery quality.

Additionally, the difference in procedure duration between groups represents a potential confounding factor that may have influenced recovery outcomes. The lack of adjustment for this variable limits the ability to fully attribute differences in recovery profile to the monitoring strategy alone.

Finally, the single-center design may limit the generalizability of the results.

Overall, these findings suggest that Entropy monitoring may contribute to improved hemodynamic stability and faster recovery during ERCP by enabling more precise titration of sedation depth, even in the absence of a significant reduction in overall adverse events. These results support its role as a valuable adjunct in clinical sedation practice. Further large-scale randomized studies are warranted to confirm these findings and better define its clinical impact.

## 5. Conclusions

This prospective observational study provides exploratory evidence regarding the clinical role of Entropy monitoring during sedation for ERCP. The primary outcome, namely the overall incidence of sedation-related adverse events, did not differ significantly between groups; therefore, this finding should not be interpreted as conclusive evidence of no effect. Within the context of these limitations, Entropy monitoring was associated with a significantly lower incidence of intraprocedural hypertensive episodes and faster postprocedural recovery, and these associations were supported by multivariable analysis adjusting for key confounders. State Entropy values correlated with clinical sedation depth, supporting its role as an objective monitoring tool. Although Entropy monitoring was not associated with a reduction in overall adverse events, it may facilitate more precise titration of sedative agents and contribute to optimized sedation management in clinical practice. These findings support the integration of Entropy monitoring in selected patients undergoing ERCP, while larger randomized studies are needed to confirm its clinical impact.

## Figures and Tables

**Table 1:** Baseline characteristics.

Parameter	Group 1 (Entropy) (*n* = 50)	Group 2 (Standard) (*n* = 50)	*p*-Value
Demographics			
Age (years), median [IQR]	70.5 [17.2]	71.5 [14.7]	0.85 ^1^
Sex (Female), *n* (%)	32 (64%)	28 (56%)	0.54 ^2^
Clinical Status			
ASA physical status, *n* (%)			0.33 ^2^
Grade II	24 (48%)	20 (40%)	
Grade III	25 (50%)	26 (52%)	
Grade IV	1 (2%)	4 (8%)	
Pulmonary disease, *n* (%)	1 (2%)	1 (2%)	1.00 ^2^
Hypertension, *n* (%)	32 (64%)	26 (52%)	0.67 ^2^
Procedure Details			
Procedure duration (min), med [IQR]	20.0 [10]	25.0 [10]	<0.001 ^1^
Malignant Diagnosis, *n* (%)	13 (26%)	18 (36%)	0.27 ^2^
Sedation Doses			
Propofol (mg), median [IQR]	110.0 [40]	100.0 [57.5]	0.68 ^1^
Fentanyl (mg), median [IQR]	0.20 [0.10]	0.22 [0.10]	0.30 ^1^
Midazolam dose (mg), mean ± SD	0.24 ± 0.65	0.14 ± 0.40	0.41 ^3^
Ketamine dose (mg), mean ± SD	1.60 ± 6.80	3.20 ± 11.68	0.66 ^3^
Interventions, *n (%)*			
Vasopressor use (ephedrine)	14 (28%)	15 (30%)	0.82 ^2^
Composite Outcome			
Desaturation (SpO_2_ < 90%)	5 (10%)	7 (14%)	0.53 ^2^
Hypotension (any episode)	14 (28%)	15 (30%)	0.49 ^2^
Hypertension (any episode)	3 (6%)	13 (26%)	0.007 ^2^
Bradycardia (HR < 50 bpm)	2 (4%)	4 (8%)	0.40 ^2^
Tachycardia (HR > 100 bpm)	1 (2%)	2 (4%)	0.55 ^2^
Cardiovascular Medications			
ACE inhibitors, *n* (%)	24 (48%)	22 (44%)	0.84 ^2^
Calcium channel blockers, *n* (%)	10 (20%)	12 (24%)	0.63 ^2^
Procedural Complexity Surrogates			
Additional cannulation attempt (at), *n* (%)	29 (58%)	38 (76%)	0.056 ^2^
Procedural Complications			
Bleeding, *n* (%)	3 (6%)	2 (4%)	1.00 ^2^
Perforation, *n* (%)	1 (2%)	2 (4%)	1.00 ^2^
Purulent bile, *n* (%)	19 (38%)	13 (26%)	0.18 ^2^

IQR: Interquartile Range; SD: standard deviation; ^1^ Mann–Whitney U test; ^2^ Chi-square test; ^3^ Student *t*-test.

**Table 2:** Clinical sedation depth and the association with adverse events.

Parameter	Adverse Event (+) (*n* = 23)	Adverse Event (−) (*n* = 27)	*p*-Value
**Age (years)**	67.0 [18]	71.0 [15]	0.21
**ASA physical status (III–IV)**	15 (65.2%)	13 (48.1%)	0.24
**Malignant Diagnosis**	7 (30.4%)	8 (29.6%)	0.95
**Procedure Duration (min)**	25.0 [10]	20.0 [5]	0.02
**Propofol Total Dose (mg)**	140.0 [70]	105.0 [40]	0.01
**MOAA/S Minimum Score**	2.0 [1]	2.0 [1]	0.85
**Minimum SE**	49.0 [10]	57.0 [8]	0.012
**Minimum RE**	52.0 [14]	61.0 [10]	0.018
**Sedation Depth**			
**Deep Sedation (MOAA/S 1–2)**	18 (78.3%)	12 (44.4%)	0.02
**Moderate Sedation (MOAA/S 3)**	5 (21.7%)	15 (55.6%)	

**Table 3:** Correlation between Entropy-derived sedation depth and sedative doses in the Entropy group.

Parameter 1	Parameter 2	r Correlation Coefficient *	*p*-Value
**SE at 3 min**	MOAA/S at 3 min	0.43	0.002
**Minimum SE**	Minimum MOAA/S	0.46	<0.001
**Minimum RE**	Minimum MOAA/S	0.43	0.002
**Minimum SE**	Hypotension	−0.28	0.04
**Minimum SE**	Desaturation	−0.21	0.13
**Minimum SE**	Bradycardia	−0.23	0.101
**Minimum RE**	Hypotension	−0.26	0.05
**Minimum RE**	Desaturation	−0.19	0.17
**Minimum RE**	Bradycardia	−0.21	0.12
**Minimum RE**	Tachycardia	−0.18	0.19
**Minimum SE**	Tachycardia	−0.20	0.15
**Minimum SE**	Propofol total dose	−0.41	0.003
**Minimum RE**	Propofol total dose	−0.38	0.006
**Minimum SE**	Fentanyl dose	−0.22	0.12
**Minimum RE**	Fentanyl dose	−0.20	0.15
**Minimum SE**	Ketamine dose	−0.18	0.19
**Minimum RE**	Ketamine dose	−0.16	0.24

* Spearman’s rank correlation coefficient. SE = State Entropy; RE = Response Entropy; MOAA/S = Modified Observer’s Assessment of Alertness/Sedation.

**Table 4:** Intraprocedural sedation depth and postprocedural recovery profile: Entropy monitoring vs. standard clinical assessment.

Parameter	Group 1 (Entropy) (*n* = 50)	Group 2 (Standard) (*n* = 50)	*p*-Value
**State Entropy**			
**Baseline (Awake), med [IQR]**	90.0 [2.7]	N/A	-
**At 3 min, med [IQR]**	70.0 [14]	N/A	-
**Minimum SE recorded, med [IQR]**	54.0 [8]	N/A	-
**MOAA/S Scale**			
**Baseline, median [IQR]**	5.0 [0]	5.0 [0]	1.00 ^1^
**At 3 min, median [IQR]**	3.0 [1]	3.0 [1]	0.74 ^1^
**Minimum MOAA/S, median [IQR]**	2.0 [1]	2.5 [1]	0.04 ^1^
**Recovery (Aldrete Score)**			
**Aldrete at 0 min, med [IQR]**	8.0 [1]	7.0 [1]	0.11 ^1^
**Aldrete at 5 min, med [IQR]**	9.0 [0]	8.0 [1]	0.003 ^1^
**Aldrete at 15 min, med [IQR]**	10.0 [0]	9.5 [1]	<0.001 ^1^

IQR—interquartile range; ^1^ Mann-Whitney U test.

## Data Availability

The data are encapsulated within this article. Further details can be obtained upon request from either the primary author or the corresponding author.

## References

[1] Chandrasekhara V., Khashab M.A., Muthusamy V.R., Acosta R.D., Agrawal D., Bruining D.H., Eloubeidi M.A., Fanelli R.D., Faulx A.L., Gurudu S.R. (2017). Adverse Events Associated with ERCP. Gastrointest. Endosc..

[2] McCarty T.R., Hathorn K.E., Creighton D.W., AlSamman M.A., Thompson C.C. (2021). Safety and Sedation-Associated Adverse Event Reporting among Patients Undergoing Endoscopic Cholangiopancreatography: A Comparative Systematic Review and Meta-Analysis. Surg. Endosc..

[3] Prosenz J., Lang R.-P., Bernhofer S., Maieron A. (2025). A Prospective Study on Incidence of Desaturations in ERCP with Non-Anesthesiologist Sedation and Adverse Event Awareness of Endoscopists. Sci. Rep..

[4] Bishay K., Meng Z.W., Khan R., Gupta M., Ruan Y., Vaska M., Iannuzzi J., O’Sullivan D.E., Mah B., Partridge A.C.R. (2025). Adverse Events Associated With Endoscopic Retrograde Cholangiopancreatography: Systematic Review and Meta-Analysis. Gastroenterology.

[5] Yang J.F., Farooq P., Zwilling K., Patel D., Siddiqui A.A. (2016). Efficacy and Safety of Propofol-Mediated Sedation for Outpatient Endoscopic Retrograde Cholangiopancreatography (ERCP). Dig. Dis. Sci..

[6] Alzanbagi A.B., Jilani T.L., Qureshi L.A., Ibrahim I.M., Tashkandi A.M.S., Elshrief E.E.A., Khan M.S., Abdelhalim M.A.H., Zahrani S.A., Mohamed W.M.K. (2022). Randomized Trial Comparing General Anesthesia with Anesthesiologist-Administered Deep Sedation for ERCP in Average-Risk Patients. Gastrointest. Endosc..

[7] Smith Z.L., Mullady D.K., Lang G.D., Das K.K., Hovis R.M., Patel R.S., Hollander T.G., Elsner J., Ifune C., Kushnir V.M. (2019). A Randomized Controlled Trial Evaluating General Endotracheal Anesthesia versus Monitored Anesthesia Care and the Incidence of Sedation-Related Adverse Events during ERCP in High-Risk Patients. Gastrointest. Endosc..

[8] Kwon M.-Y., Lee S.-Y., Kim T.-Y., Kim D.K., Lee K.-M., Woo N.-S., Chang Y.-J., Lee M.A. (2012). Spectral Entropy for Assessing the Depth of Propofol Sedation. Korean J. Anesthesiol..

[9] Janik L.S., Stamper S., Vender J.S., Troianos C.A. (2022). Pro-Con Debate: Monitored Anesthesia Care Versus General Endotracheal Anesthesia for Endoscopic Retrograde Cholangiopancreatography. Anesth. Analg..

[10] Azimaraghi O., Bilal M., Amornyotin S., Arain M., Behrends M., Berzin T.M., Buxbaum J.L., Choice C., Fassbender P., Sawhney M.S. (2023). Consensus Guidelines for the Perioperative Management of Patients Undergoing Endoscopic Retrograde Cholangiopancreatography. Br. J. Anaesth..

[11] Applegate R.L., Lenart J., Malkin M., Meineke M.N., Qoshlli S., Neumann M., Jacobson J.P., Kruger A., Ching J., Hassanian M. (2016). Advanced Monitoring Is Associated with Fewer Alarm Events During Planned Moderate Procedure-Related Sedation: A 2-Part Pilot Trial. Anesth. Analg..

[12] Conway A., Sutherland J. (2016). Depth of Anaesthesia Monitoring during Procedural Sedation and Analgesia: A Systematic Review and Meta-Analysis. Int. J. Nurs. Stud..

[13] Stasiowski M.J., Starzewska M., Niewiadomska E., Król S., Marczak K., Żak J., Pluta A., Eszyk J., Grabarek B.O., Szumera I. (2021). Adequacy of Anesthesia Guidance for Colonoscopy Procedures. Pharmaceuticals.

[14] (2018). Practice Guidelines for Moderate Procedural Sedation and Analgesia 2018: A Report by the American Society of Anesthesiologists Task Force on Moderate Procedural Sedation and Analgesia, the American Association of Oral and Maxillofacial Surgeons, American College of Radiology, American Dental Association, American Society of Dentist Anesthesiologists, and Society of Interventional Radiology *. Anesthesiology.

[15] Jun M.R., Yoo J.H., Park S.Y., Na S., Kwon H., Nho J.-H., Kim S.I. (2019). Assessment of Phase-Lag Entropy, a New Measure of Electroencephalographic Signals, for Propofol-Induced Sedation. Korean J. Anesthesiol..

[16] Schmidt G.N., Bischoff P., Standl T., Hellstern A., Teuber O., Schulte Am Esch J. (2004). Comparative Evaluation of the Datex-Ohmeda S/5 Entropy Module and the Bispectral Index® Monitor during Propofol–Remifentanil Anesthesia. Anesthesiology.

[17] Huang L., Liu L., Lu Y., Zhuang M., Dou W., Liu H., Ji F., Peng K. (2025). Assessing Sedation Depth with PSI in Elderly ERCP Patients: A Prospective Cohort Study. Clin. Interv. Aging.

[18] Bein B. (2006). Entropy. Best Pract. Res. Clin. Anaesthesiol..

[19] Dinu A.R., Rogobete A.F., Popovici S.E., Bedreag O.H., Papurica M., Dumbuleu C.M., Velovan R.R., Toma D., Georgescu C.M., Trache L.I. (2020). Impact of General Anesthesia Guided by State Entropy (SE) and Response Entropy (RE) on Perioperative Stability in Elective Laparoscopic Cholecystectomy Patients—A Prospective Observational Randomized Monocentric Study. Entropy.

[20] Paspatis G., Chainaki I., Manolaraki M., Vardas E., Theodoropoulou A., Tribonias G., Konstantinidis K., Karmiris K., Chlouverakis G. (2009). Efficacy of Bispectral Index Monitoring as an Adjunct to Propofol Deep Sedation for ERCP: A Randomized Controlled Trial. Endoscopy.

[21] Von Delius S., Salletmaier H., Meining A., Wagenpfeil S., Saur D., Bajbouj M., Schneider G., Schmid R., Huber W. (2012). Bispectral Index Monitoring of Midazolam and Propofol Sedation during Endoscopic Retrograde Cholangiopancreatography: A Randomized Clinical Trial (the EndoBIS Study). Endoscopy.

[22] Jokelainen J., Mustonen H., Kylänpää L., Udd M., Lindström O., Pöyhiä R. (2018). Assessment of Sedation Level for Endoscopic Retrograde Cholangiopancreatography–a Prospective Validation Study. Scand. J. Gastroenterol..

[23] Gemma M., Pennoni F., Tritto R., Agostoni M. (2021). Risk of Adverse Events in Gastrointestinal Endoscopy: Zero-Inflated Poisson Regression Mixture Model for Count Data and Multinomial Logit Model for the Type of Event. PLoS ONE.

[24] Punjasawadwong Y., Phongchiewboon A., Bunchungmongkol N. (2014). Bispectral Index for Improving Anaesthetic Delivery and Postoperative Recovery. Cochrane Database Syst. Rev..

